# Waveform-Level Validation of Continuous Shank-to-Vertical Angle Measurement Using a Wearable Posture Sensor with Independent Video-Based Gait Event Detection

**DOI:** 10.3390/s26144392

**Published:** 2026-07-10

**Authors:** Souji Tanaka

**Affiliations:** Department of Prosthetics, Orthotics and Assistive Technology, Faculty of Rehabilitation, Niigata University of Health and Welfare, 1398 Shimami-cho, Kita-ku, Niigata 950-3198, Japan; soji-tanaka@nuhw.ac.jp

**Keywords:** wearable sensor, shank-to-vertical angle, gait analysis, ankle–foot orthosis, motion capture

## Abstract

Accurate evaluation of the shank-to-vertical angle (SVA) is important for optimizing lower-limb alignment during gait, particularly during ankle–foot orthosis (AFO) tuning. This study investigated the validity of a wearable posture sensor for continuous SVA measurement during walking using an optical motion capture system as the reference standard. Nine healthy adults participated, and 88 gait cycles were analyzed. SVA was measured using a shank-mounted wearable sensor and a three-dimensional motion capture system. Gait events for the wearable sensor were identified independently using synchronized tablet-based video recordings rather than inertial signals. Agreement was evaluated at the gait-cycle and participant levels using waveform correlation, mean bias, root mean square error (RMSE), and Bland–Altman analysis. Across the full gait cycle, the trial-level waveform correlation was 0.935 ± 0.064 and the RMSE was 9.74 ± 3.71°. During the individually identified stance phase (mean toe-off, 63.67 ± 1.78% of the gait cycle), waveform correspondence increased to 0.990 ± 0.010 and the RMSE decreased to 6.78 ± 3.51°. Participant-level estimates were similar. For SVA range, participant-level Bland–Altman analysis yielded a bias of −5.58° with 95% limits of agreement from −14.01° to 2.86°, and repeated-measures analysis produced similar estimates. Temporal error analysis showed smaller and more stable deviations during the majority of the stance phase than during swing, with larger deviations in late swing. These findings support the potential use of the wearable posture sensor as a targeted tool for stance-phase SVA assessment, although further validation of pathological gait and actual orthotic tuning is required.

## 1. Introduction

Wearable inertial sensors are increasingly being adopted for quantitative gait analysis because they enable biomechanical measurements to be performed outside specialized motion analysis laboratories. Although optical motion capture systems remain the reference standard for evaluating lower-limb kinematics during walking, their high cost, technical complexity, and limited accessibility restrict routine implementation in clinical practice [1,2,3]. Recent systematic reviews have demonstrated that inertial measurement unit (IMU)-based systems can provide valid kinematic measurements when compared with gold-standard motion capture systems, particularly for many sagittal-plane lower-limb variables [4,5]. In parallel, smartphone/video-based gait analysis and AI-based markerless pose-estimation methods have emerged as accessible alternatives for clinical and field-based gait assessment [6,7,8,9]. These approaches offer important advantages in terms of cost, accessibility, and ease of implementation; however, their accuracy and applicability may depend on camera position, body orientation, task characteristics, algorithm performance, and potential occlusion from clothing or orthotic devices.

Among clinically relevant gait parameters, the shank-to-vertical angle (SVA) is widely recognized as an important indicator of sagittal-plane alignment during walking. SVA represents the orientation of the shank relative to the vertical axis and is closely associated with the position of the ground reaction force vector relative to the knee joint center during stance [10]. Appropriate forward progression of the shank contributes to stable rocker function, efficient forward advancement of the body, and adequate knee stability during gait. Previous studies have demonstrated that AFO-footwear combination tuning influences knee kinematics and kinetics, and that SVA is a useful parameter for evaluating AFO tuning [11,12].

However, orthotic alignment influences not only discrete gait events but also the continuous progression of shank inclination throughout the stance phase. In particular, the loading response phase plays a critical role in heel rocker function and in regulating the position of the ground reaction force vector relative to the knee joint [10]. Previous studies have also shown that AFO mechanical properties and alignment adjustments influence ankle and knee mechanics across multiple phases of stance through rocker-based kinetic interactions [13,14,15]. These findings suggest that evaluations limited to a single discrete time point, such as midstance, may not fully capture clinically meaningful alignment behavior during orthotic tuning.

Wearable posture sensors have been investigated as practical tools for estimating segment orientation during walking [1,3]. More recent studies have applied shank-mounted sensors to estimate SVA; however, many previous investigations primarily focused on discrete gait events or selected stance-phase parameters rather than continuous waveform agreement throughout the stance phase. For example, de Jong et al. evaluated IMU-based SVA measurements primarily at midstance, whereas other studies focused on selected stance-phase events or peak values [16]. In addition, several previous approaches relied on inertial signals for both gait event detection and orientation estimation, creating methodological dependence because both outcomes were derived from the same sensor signals [17,18].

Despite recent advances in wearable sensors, video-based gait analysis, and AI-based markerless methods, few studies have evaluated agreement across continuous stance-phase SVA waveforms in a manner directly applicable to orthotic tuning. Because AFO–footwear combination tuning is an iterative process that requires repeated adjustment and reassessment [11], a practical system capable of providing continuous stance-phase SVA feedback outside laboratory environments would be valuable for clinical application. In this context, a single shank-mounted wearable posture sensor combined with independent video-based gait event detection may provide a clinically feasible approach for targeted stance-phase SVA assessment.

Therefore, the purpose of this study was to investigate the waveform-level validity of continuous SVA measurement using a wearable posture sensor combined with independent video-based gait event detection during walking. The study specifically evaluated agreement between wearable sensor measurements and optical motion capture measurements across the gait cycle, with particular emphasis on the clinically relevant stance phase.

## 2. Materials and Methods

### 2.1. Participants

Nine healthy adults participated in this study. All participants were able to walk independently and reported no neurological or musculoskeletal disorders that could affect gait performance. Participant characteristics are summarized in Table 1.

Written informed consent was obtained from all participants prior to study participation.

### 2.2. Instrumentation

**Figure 1:**
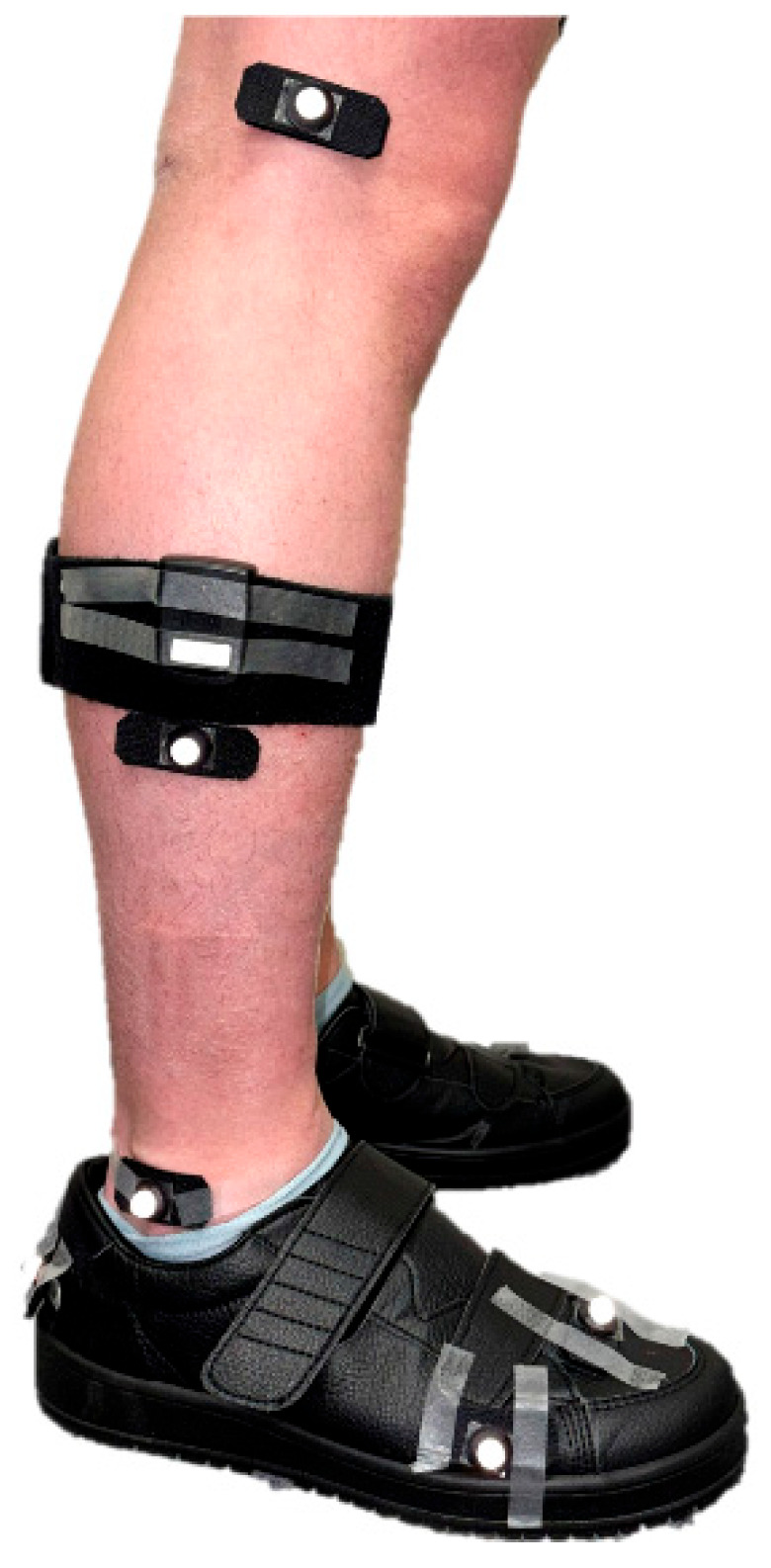
Placement and fixation of the wearable posture sensor on the shank segment. The sensor was attached to the lateral aspect of the shank and secured using an elastic Velcro strap, double-sided adhesive tape, and additional surgical tape to minimize relative motion between the sensor and skin during walking.

Shank orientation was measured using a wearable posture sensor (Teijin Frontier Sensing Co., Ltd., Fukuoka, Japan) sampled at 60 Hz. The sensor was attached to the lateral aspect of the shank at the proximal one-third of the line connecting the fibular head and lateral malleolus (Figure 1). This location corresponded to the typical strap height used during AFO alignment assessment. The sensor was secured using an elastic Velcro strap, double-sided adhesive tape, and additional surgical tape to minimize relative motion between the sensor and skin during walking (Figure 1).

Reference kinematic data were collected using a three-dimensional optical motion capture system (Vicon Motion Systems Ltd., Oxford, UK) equipped with 12 infrared cameras operating at 250 Hz. Ground reaction forces were simultaneously recorded using six force plates (AMTI, Watertown, MA, USA) sampled at 1000 Hz.

Reflective markers were placed according to the Plug-in Gait lower-body model (Figure 1). Additional foot markers were included to improve the overall tracking robustness of the lower-limb marker set during gait.

### 2.3. Experimental Procedure

Participants walked at a self-selected comfortable speed along a level walkway while wearing standardized measurement shoes (Vstep, Moonstar Co., Kurume, Japan).

Prior to sensor attachment, a primary calibration procedure was performed to define the sensor coordinate axes relative to the global reference frame according to the manufacturer’s standard initialization procedure. After sensor placement, a secondary alignment calibration was applied to align the sensor coordinate system with the anatomical sagittal plane of the shank segment. During this procedure, participants were instructed to stand in a relaxed upright posture while the sensor processing algorithm aligned the sensor coordinate system with the anatomical sagittal plane of the shank segment.

Initial-contact timing for the motion capture data was identified using vertical ground reaction force signals in combination with marker-based kinematic information, whereas gait events for the wearable posture sensor were determined using synchronized tablet-based video recordings captured at 30 Hz. Video recordings were aligned with the motion capture data using a manual frame-matching procedure based on initial contact.

### 2.4. Data Processing

Reference SVA was calculated as the sagittal-plane inclination of the shank segment relative to the global vertical axis using the vector connecting the ankle and knee joint centers, consistent with established clinical definitions [10]. Joint centers were approximated from marker positions based on a standard lower-limb marker set, and the shank segment vector was constructed from knee and ankle marker positions.

Initial contact was identified by combining vertical ground reaction force data and marker-based kinematic methods [19], whereas gait events for the wearable posture sensor were determined independently as described in Section 2.3. Gait cycles were defined from initial contact to the subsequent initial contact and were time-normalized to 0–100% of the gait cycle.

Toe-off was identified individually for each gait cycle from the vertical ground reaction force signals using a 20 N threshold. The individually identified toe-off percentage was used to define the stance-phase interval for both the optical motion capture and wearable sensor waveforms, rather than applying a fixed 60% cutoff to all gait cycles.

Marker trajectories and ground reaction force signals were filtered within the motion capture system (Nexus software, version 2.12, Vicon Motion Systems Ltd.) prior to export. Ground reaction force signals were low-pass filtered using a fourth-order Butterworth filter with a cutoff frequency of 50 Hz. No additional filtering was applied to the motion capture kinematic data during post-processing. Wearable sensor signals were processed using a fourth-order zero-phase Butterworth low-pass filter with a cutoff frequency of 6 Hz.

Each gait cycle was resampled to 101 points (0–100%) using piecewise cubic Hermite interpolation to enable point-by-point waveform comparison between the two systems.

All data processing and analyses were performed using custom-written scripts in MATLAB R2025a (MathWorks, Natick, MA, USA).

### 2.5. Quality Control

Gait cycles outside 0.75–2.0 s or walking speeds outside 0.6–1.8 m/s were excluded. Trials affected by marker occlusion or incomplete force plate contact were excluded according to predefined quality control criteria. After application of these criteria, 88 gait cycles were included in the final analysis.

### 2.6. Statistical Analysis

Agreement between wearable sensor-derived SVA and motion capture-derived SVA was evaluated using Pearson correlation coefficients, mean bias, and root mean square error (RMSE). Differences were defined as wearable posture sensor values minus optical motion capture values. Waveform-level correlation, bias, and RMSE were calculated for each gait cycle for both the full gait cycle and the individually identified stance phase. Trial-level results were retained to characterize gait-cycle variability, and participant-level mean values were additionally calculated to confirm that the findings were not driven by repeated observations within participants.

For group waveform summaries, gait-cycle waveforms were first averaged within each participant and then averaged across participants so that each participant contributed equally. The mean individually identified toe-off percentage was displayed as the stance-to-swing transition in the group waveform and temporal error figures.

Agreement in SVA range was assessed using Bland–Altman analysis. Participant-level mean SVA range values were used for the primary analysis so that each participant represented an independent observational unit. The original trial-level analysis was retained as a descriptive comparison. In addition, a repeated-measures Bland–Altman analysis based on variance components of the paired differences was performed to account for gait cycles nested within participants [20]. The present analysis focused on descriptive waveform-level agreement and potential clinical applicability rather than inferential testing of waveform differences; therefore, descriptive agreement metrics were selected instead of inferential waveform statistics such as statistical parametric mapping.

**Figure 2:**
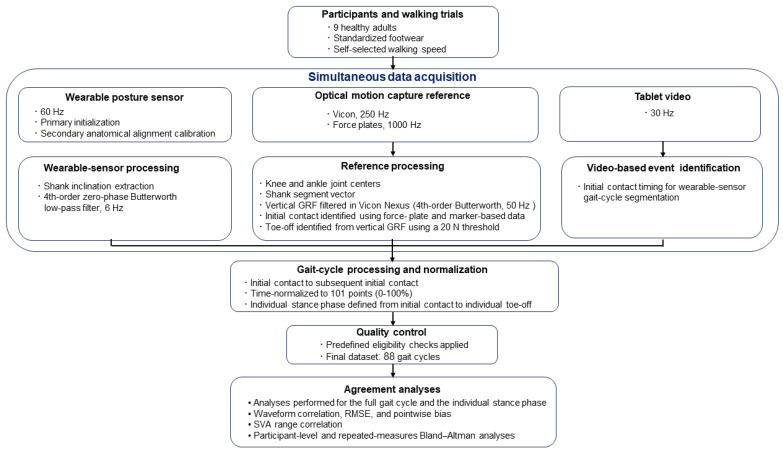
Methodological workflow for validation of continuous shank-to-vertical angle (SVA) measurement. Wearable posture sensor data, optical motion capture and force-plate data, and tablet video were acquired simultaneously. Wearable-sensor gait cycles were segmented using initial-contact timing identified from synchronized tablet video, independently of the wearable orientation signal. Reference SVA was calculated from the shank segment defined by the knee and ankle joint centers. Initial contact for the reference data was identified using force-plate and marker-based information, and individual toe-off was identified from the vertical ground reaction force using a 20-N threshold. Gait cycles were time-normalized to 101 points, and the individual stance phase was defined from initial contact to toe-off. After quality control, 88 gait cycles were included in waveform- and SVA-range agreement analyses.

The overall methodological workflow, from simultaneous data acquisition to gait-cycle processing, quality control, and agreement analyses, is summarized in Figure 2.

## 3. Results

### 3.1. Agreement Across the Full Gait Cycle

**Figure 3:**
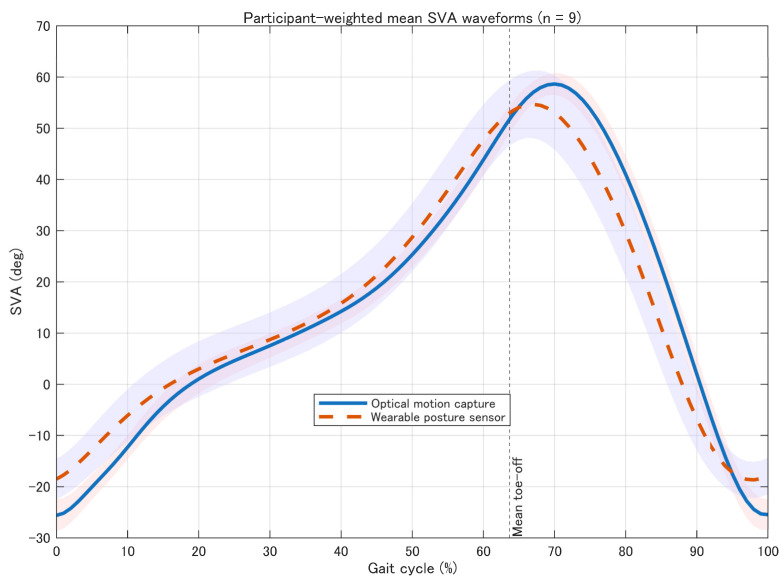
Participant-weighted mean shank-to-vertical angle (SVA) waveforms obtained from the optical motion capture system and wearable posture sensor across the gait cycle. Gait-cycle waveforms were first averaged within each participant and then averaged across participants (*n* = 9). Shaded regions indicate between-participant standard deviations. The vertical dashed line denotes the mean individually identified toe-off (63.67% of the gait cycle).

Across the full gait cycle, the wearable posture sensor showed moderate-to-strong waveform correspondence with optical motion capture. At the trial level, the mean Pearson correlation coefficient was 0.935 ± 0.064, the RMSE was 9.74 ± 3.71°, and the mean pointwise bias was −0.02 ± 5.14°. Participant-level mean estimates were closely comparable (r = 0.936 ± 0.024; RMSE = 9.71 ± 1.77°; bias = 0.00 ± 5.18°), indicating that the trial-level findings were not materially influenced by repeated gait cycles within participants (Table 2, Figure 3).

### 3.2. Agreement During the Stance Phase

Agreement improved when the analysis was restricted to the individually identified stance phase. The mean toe-off timing was 63.67 ± 1.78% of the gait cycle. At the trial level, stance-phase waveform correlation was r = 0.990 ± 0.010, RMSE was 6.78 ± 3.51°, and mean pointwise bias was 3.33 ± 5.98°. Participant-level estimates were nearly identical (r = 0.990 ± 0.006; RMSE = 6.76 ± 2.09°; bias = 3.33 ± 4.93°), supporting the robustness of the trial-level findings (Table 2). Analyses based on a fixed 60% cutoff and on individual toe-off produced closely comparable estimates, indicating that use of individualized toe-off improved the physiological definition of stance without materially changing the principal findings.

**Figure 4:**
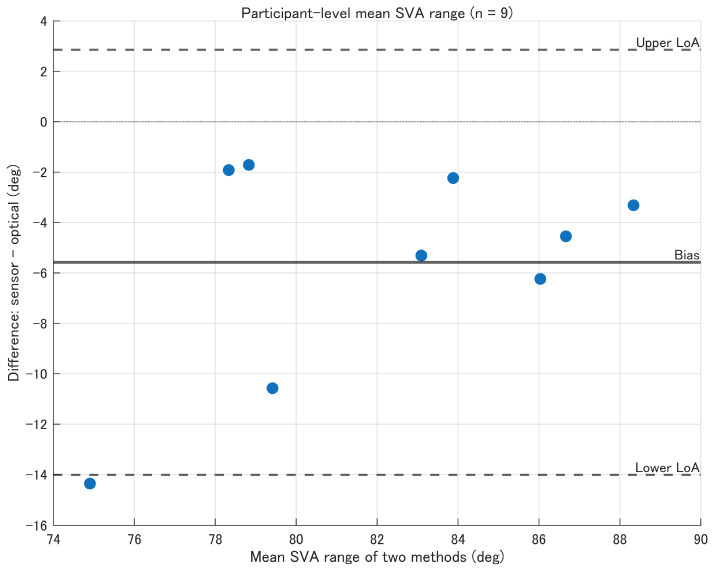
Bland–Altman agreement of participant-level mean SVA range between the wearable posture sensor and optical motion capture system (*n* = 9). Differences were calculated as wearable posture sensor values minus optical motion capture values. The solid line indicates the mean bias, and the dashed lines indicate the 95% limits of agreement.

For SVA range, the participant-level Bland–Altman analysis yielded a mean bias of −5.58° with 95% limits of agreement from −14.01° to 2.86° (*n* = 9). The participant-level Bland–Altman plot is shown in Figure 4. The repeated-measures analysis, which retained all 88 gait cycles while accounting for clustering within participants, produced closely comparable estimates (bias, −5.61°; 95% limits of agreement, −14.75° to 3.52°). The trial-level correlation between SVA range values remained moderate (r = 0.67), indicating a moderate association in the magnitude of cycle-to-cycle SVA excursion rather than point-by-point waveform similarity.

Accordingly, the lower SVA-range correlation does not contradict the high stance-phase waveform correlation. These analyses address different properties: the waveform correlation quantifies similarity in temporal shape within each gait cycle, whereas the SVA-range analyses evaluate between-cycle and between-participant agreement in angular excursion.

### 3.3. Temporal Distribution of Measurement Error

**Figure 5:**
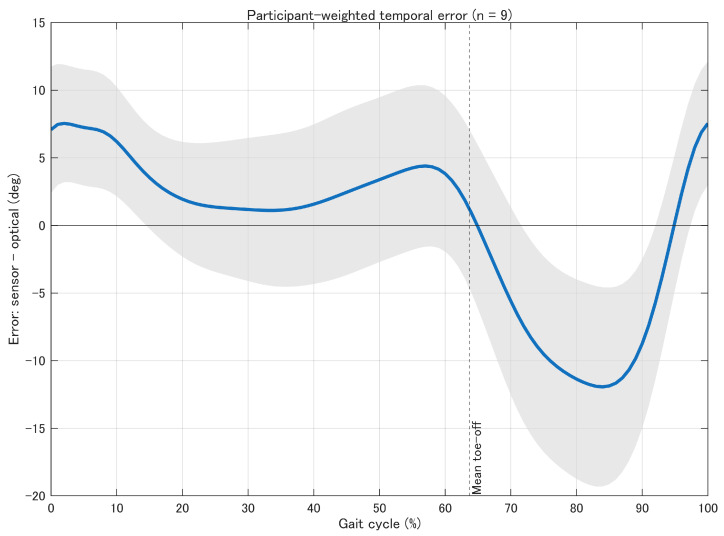
Participant-weighted temporal distribution of SVA error (wearable posture sensor minus optical motion capture) across the gait cycle. Gait-cycle error waveforms were first averaged within each participant and then averaged across participants (*n* = 9). The solid line represents the mean error, and the shaded region indicates ±1 between-participant standard deviation. The vertical dashed line denotes the mean individually identified toe-off (63.67% of the gait cycle).

Temporal error analysis demonstrated that measurement deviations were not uniformly distributed throughout the gait cycle. The temporal distribution of SVA error across the gait cycle is shown in Figure 5. The participant-weighted mean error was generally smaller and more stable during much of the stance phase than during the swing phase, although a positive offset remained during portions of stance. This pointwise error waveform should not be interpreted as equivalent to RMSE, mean bias, or Bland–Altman limits of agreement, because these metrics summarize different aspects and levels of measurement error.

Larger deviations were observed during the swing phase, particularly in late swing. The close correspondence between trial-level and participant-level summary estimates indicates that the principal temporal error pattern was not attributable to unequal numbers of gait cycles or within-participant clustering.

## 4. Discussion

This study investigated the waveform-level validity of continuous shank-to-vertical angle (SVA) measurement using a single wearable posture sensor with independent video-based gait event detection. Across the full gait cycle, the sensor showed moderate-to-strong waveform correspondence with optical motion capture, whereas agreement was substantially stronger during the individually identified stance phase. Larger deviations were concentrated mainly during swing, particularly late swing. Because stance is the principal interval used to evaluate shank progression and ground reaction force alignment during orthotic tuning, these findings support the potential use of the sensor as a targeted tool for stance-phase SVA assessment rather than as a substitute for highly accurate full-cycle motion capture.

To clarify the methodological position of the proposed system, Table 3 summarizes contemporary motion analysis methods used in clinical gait evaluation. Direct numerical comparison across these approaches remains difficult because published studies differ in target variables, participant populations, movement tasks, camera or sensor configurations, algorithms, and reported accuracy metrics. Optical motion capture remains the reference standard for laboratory-based gait analysis but is limited by high cost, technical complexity, and restricted clinical accessibility. Wearable IMU-based systems have shown generally favorable agreement with optical motion capture for many sagittal-plane kinematic variables [4,5], while smartphone/video-based and AI-based markerless methods are emerging as accessible alternatives for gait assessment [6,7,8,9]. However, AFO tuning requires not only general gait screening or discrete spatiotemporal parameters but also continuous assessment of shank progression during the weight-bearing stance phase. Single-camera video-based methods may be affected by perspective, body orientation, and possible occlusion from clothing or orthotic devices, whereas conventional multi-IMU systems provide more comprehensive kinematics but require multiple sensors and calibration procedures [21,22]. The proposed single-sensor approach is therefore positioned as a targeted method for continuous stance-phase SVA assessment with minimal setup burden, which may be advantageous for repeated, iterative AFO tuning.

Independent video-based gait event detection separated temporal segmentation from orientation estimation. Previous IMU validation studies have frequently derived both gait events and orientation estimates from the same inertial signals [17,18]. In the present study, initial-contact timing for the wearable sensor was instead identified from synchronized tablet video, thereby avoiding reliance on the wearable orientation signal for gait-cycle segmentation. However, the present study did not directly compare the accuracy of video-based and IMU-based gait event detection. Therefore, these findings should not be interpreted as demonstrating that video-based event detection is more accurate or superior to IMU-based methods.

The dynamic accuracy of the wearable posture sensor should be interpreted by distinguishing waveform shape, pointwise angular error, and agreement in SVA range. The full-cycle pointwise bias was close to zero, whereas the stance-phase pointwise bias was approximately +3.33°, indicating a positive offset during portions of stance despite the high waveform correlation. In contrast, the Bland–Altman analysis of SVA range showed a negative bias of approximately −5.6°, indicating that the wearable sensor tended to underestimate the overall angular excursion. These findings are not contradictory because they describe different properties of agreement. The remaining offsets may have been influenced by initial sensor attachment angle, anatomical alignment calibration, sensor-to-segment orientation, and phase-dependent soft-tissue motion.

Several methodological features may have contributed to the strong stance-phase waveform correspondence observed in this study. First, sensor-axis initialization was combined with an additional anatomical alignment calibration procedure to align the sensor coordinate system with the sagittal plane of the shank segment. Because SVA estimation depends on accurate alignment between the sensor and segment coordinate systems, this step likely reduced axis misalignment and cross-talk [23,24]. Second, temporal alignment was achieved using tablet-based video recordings rather than hardware triggering, which may improve practical applicability in clinical settings where direct electrical synchronization is often unavailable. Third, magnetometer signals were excluded from orientation estimation to improve robustness in magnetically disturbed indoor environments. Although magnetometer-free approaches have been reported to provide stable orientation estimation under controlled conditions [25], heading drift was not directly quantified and remains a potential source of error.

Temporal error analysis showed that measurement deviations were not uniformly distributed throughout the gait cycle. Errors were generally smaller and more stable during much of stance, although a positive offset remained during portions of this phase. Larger negative deviations emerged after toe-off and were most pronounced during late swing, which is consistent with soft-tissue artifact effects associated with rapid limb motion and displacement of surface-mounted sensors or markers relative to the underlying skeletal structures during high-velocity phases of gait [26]. This phase-dependent pattern explains why full-cycle RMSE was larger than stance-phase RMSE despite relatively strong overall waveform correspondence.

The clinical significance of the observed errors should be interpreted cautiously in relation to the intended application of the system. In AFO tuning, shank progression during the weight-bearing stance phase is clinically relevant because it reflects the interaction between the orthosis, footwear, and ground reaction force [10,12]. Previous studies have shown that SVA can respond to controlled changes in AFO-footwear alignment, including alterations in heel height [12,27]. However, a universally accepted clinically acceptable measurement-error threshold for continuous SVA waveforms during AFO tuning has not been established. In the present study, the stance-phase RMSE was 6.78 ± 3.51° at the trial level and 6.76 ± 2.09° at the participant level. Therefore, the present results do not demonstrate that the sensor can reliably detect small changes resulting from fine orthotic adjustments, nor do they establish interchangeability with optical motion capture. Rather, the high waveform correspondence suggests that the sensor may be useful for characterizing the overall temporal pattern of stance-phase shank progression. Its responsiveness and ability to detect clinically meaningful changes during actual AFO tuning should be evaluated directly in future studies involving AFO users.

The close correspondence between trial-level and participant-level estimates strengthens the interpretation of the waveform findings. In addition, participant-level and repeated-measures Bland–Altman analyses yielded nearly identical estimates for SVA range, indicating that the principal agreement results were not materially affected by repeated gait cycles within participants. Retaining the trial-level analysis preserved information regarding cycle-to-cycle variability, whereas the participant-level and repeated-measures analyses provided confirmation that within-participant clustering did not substantially alter the conclusions.

Although advanced mathematical models may further improve wearable sensor-based kinematic estimation, the present study intentionally focused on validating a simple single-sensor approach without incorporating complex modeling. Multibody optimization, machine learning algorithms, and advanced sensor-fusion methods may reduce kinematic estimation errors and soft-tissue artifacts [24,28]. Repeated paired measurements from the same individual could also be used to develop subject-specific calibration or learning-based error-correction models, potentially reducing systematic and phase-dependent errors. However, this approach was beyond the scope of the present validation study and would require separate training data for each individual. These individualized and model-based approaches, however, often require additional computational processing, subject-specific modeling, prior training data, or more complex calibration procedures, which may reduce the simplicity and immediacy required for iterative clinical AFO tuning. Future studies should examine whether lightweight mathematical models or optimized sensor-fusion approaches can improve swing-phase and full-cycle accuracy while preserving minimal setup burden and immediate interpretability.

## 5. Limitations

Several limitations should be considered when interpreting the present findings.

First, validation was performed in healthy participants. Therefore, the findings may not be directly generalizable to clinical populations exhibiting pathological gait patterns, altered shank kinematics, reduced walking speed, increased gait variability, or the use of assistive devices. In particular, individuals who use ankle–foot orthoses may show atypical limb trajectories and additional sources of measurement error related to orthotic components, straps, or altered soft-tissue motion. Accordingly, the present findings should be interpreted as proof-of-concept evidence in healthy adults rather than as direct validation of clinical performance in individuals with pathological gait or in actual AFO users. Future studies should evaluate the proposed system in individuals with neurological or musculoskeletal gait impairments and in patients wearing AFOs during actual orthotic tuning.

Second, although agreement was higher during the individually identified stance phase than across the full gait cycle, the observed stance-phase RMSE indicates that the proposed system should not be interpreted as a substitute for optical motion capture when precise absolute-angle measurements are required. Larger phase-dependent errors were observed during swing, particularly in late swing. The proposed system should therefore be regarded as a targeted tool for characterizing stance-phase SVA progression. Furthermore, because a clinically acceptable error threshold for continuous SVA measurement during AFO tuning has not been established, the ability of the present system to detect small changes resulting from fine orthotic adjustments remains uncertain. Responsiveness should therefore be evaluated directly under controlled AFO-tuning conditions. Further refinement of calibration procedures, sensor fixation, and signal processing may be necessary to improve absolute agreement and full-cycle accuracy.

Third, the direction and magnitude of bias depended on the outcome being evaluated. The full-cycle pointwise bias was close to zero, whereas a positive pointwise bias was observed during stance and the wearable sensor underestimated full-cycle SVA range by approximately 5.6°. These findings may reflect participant-specific differences in sensor attachment, anatomical alignment calibration, and sensor-to-segment orientation, as well as phase-dependent waveform differences. Future studies should examine calibration procedures that reduce inter-participant offset variability and improve both absolute-angle and range agreement.

Fourth, gait event detection for the wearable sensor was based on manual identification from 30 Hz tablet video. Although this approach provided temporal segmentation independent of the wearable sensor signals, small frame-matching errors cannot be completely excluded. In addition, the present study did not directly compare video-based event detection with IMU-based event-detection algorithms; therefore, no conclusion can be drawn regarding the relative accuracy or superiority of these approaches. Higher-frame-rate video, automated video-based event detection, synchronized external triggering, and direct method comparisons should be examined in future studies.

Fifth, the present study intentionally evaluated a simple single-sensor approach without incorporating advanced mathematical models, machine learning algorithms, or multibody optimization. Such methods may improve full-cycle kinematic accuracy and reduce soft-tissue artifact effects, particularly during the swing phase. However, the purpose of this study was to assess the baseline concurrent validity of a simple system intended for clinical SVA measurement. Future work should explore whether lightweight mathematical models or optimized sensor-fusion algorithms can improve accuracy while preserving the simplicity, immediacy, and minimal setup burden required for routine clinical AFO tuning.

Finally, the relatively small sample of nine healthy participants reflects the proof-of-concept nature of this study. Multiple gait cycles improved characterization of cycle-to-cycle variability but did not increase the number of independent participants. Although participant-level and repeated-measures analyses yielded closely comparable estimates, the participant-level Bland–Altman limits of agreement remain uncertain because they were derived from only nine participants. Further validation in larger and more diverse cohorts is required to obtain more precise agreement estimates and confirm the robustness and generalizability of the proposed approach.

## 6. Conclusions

This study demonstrated that a single shank-mounted wearable posture sensor captured the temporal pattern of SVA measured by optical motion capture, with particularly high waveform correspondence during the individually identified stance phase. Trial-level and participant-level estimates were closely comparable, and participant-level and repeated-measures Bland–Altman analyses of SVA range yielded similar results, supporting the robustness of the findings. Nevertheless, the sensor underestimated SVA range on average, and larger phase-dependent errors were observed during swing, particularly in late swing. Accordingly, the proposed system should not be considered a substitute for optical motion capture when precise full-cycle kinematic measurements are required. Rather, it may offer a feasible and accessible approach for targeted assessment of stance-phase shank progression during iterative AFO tuning. These findings should be regarded as proof-of-concept evidence in healthy adults, and further validation in individuals with pathological gait and in actual orthotic tuning conditions is warranted.

## Figures and Tables

**Table 1:** Participant characteristics.

Variable	Mean ± SD
Age (years)	20.1 ± 0.5
Height (cm)	170.2 ± 6.5
Body mass (kg)	62.4 ± 5.7
Self-selected walking speed (m/s)	1.29 ± 0.13
Number of analyzed gait cycles	88

**Table 2:** Agreement metrics.

Metric	Full Gait Cycle (0–100%)	Individual Stance Phase
Waveform correlation, trial level (r)	0.935 ± 0.064	0.990 ± 0.010
Waveform correlation, participant level (r)	0.936 ± 0.024	0.990 ± 0.006
RMSE, trial level (°)	9.74 ± 3.71	6.78 ± 3.51
RMSE, participant level (°)	9.71 ± 1.77	6.76 ± 2.09
Mean bias, trial level (°)	−0.02 ± 5.14	3.33 ± 5.98
Mean bias, participant level (°)	0.00 ± 5.18	3.33 ± 4.93

**Table 3:** Comparison of motion analysis methods for clinical gait evaluation.

Method	Equipment and Output	Feasibility/Accuracy	Main Advantages	Main Limitations	References
Optical motion capture	Multiple infrared cameras, reflective markers, and dedicated laboratory space; provides 3D joint and segment kinematics	Reference standard; low clinical feasibility	Comprehensive 3D kinematic assessment with high temporal and spatial resolution	Expensive, laboratory-bound, time-consuming setup; requires trained personnel	[1,2,4,5]
Smartphone/video-based gait analysis	Smartphone or digital camera with video processing software; provides spatiotemporal parameters and selected 2D kinematics	High feasibility; accuracy varies by camera position, task, algorithm, and reference system	Low cost, widely available, non-contact, suitable for basic screening	Susceptible to perspective distortion and camera placement; limited continuous angular waveform assessment	[6,7]
AI-based markerless pose estimation	Video camera or smartphone with markerless pose-estimation algorithm; provides 2D or 3D joint-center trajectories and kinematics	Moderate to high feasibility; accuracy varies by model, body segment, task, and reference system	Markerless and unobtrusive; estimates multiple body landmarks without physical sensors	Affected by camera view, model performance, atypical gait, and occlusion from clothing or orthotic devices	[7,8,9]
Conventional multi-IMU gait analysis	Multiple IMUs with dedicated software and sensor-to-segment calibration; provides lower-limb kinematics and spatiotemporal parameters	Moderate feasibility; generally favorable for many sagittal-plane measures but varies by joint, task, and calibration	Portable; enables continuous kinematic assessment outside motion laboratories	Multiple sensors; setup and calibration burden; potential drift, soft-tissue artifact, and magnetic disturbance	[4,5,23,24]
Proposed single wearable posture sensor	Single shank-mounted wearable posture sensor with tablet-based video event detection; provides continuous sagittal-plane SVA waveform	High feasibility; individual stance-phase RMSE: 6.78 ± 3.51° at the trial level and 6.76 ± 2.09° at the participant level; larger errors across the full gait cycle	Minimal setup; directly targets SVA for iterative AFO tuning; independent video-based gait event detection	Limited to sagittal-plane shank orientation; validated in healthy participants; swing-phase and full-cycle accuracy require improvement	Present study

## Data Availability

The data presented in this study are available upon reasonable request from the corresponding author.

## Abbreviations

The following abbreviations are used in this manuscript:

AFO	Ankle–Foot Orthosis
IMU	Inertial Measurement Unit
LoA	Limits of Agreement
RMSE	Root Mean Square Error
SVA	Shank-to-Vertical Angle
